# Interactions between fecal gut microbiome, enteric pathogens, and energy regulating hormones among acutely malnourished rural Gambian children

**DOI:** 10.1016/j.ebiom.2021.103644

**Published:** 2021-10-22

**Authors:** Helen M. Nabwera, Josh L. Espinoza, Archibald Worwui, Modupeh Betts, Catherine Okoi, Abdul K. Sesay, Rowan Bancroft, Schadrac C. Agbla, Sheikh Jarju, Richard S. Bradbury, Mariama Colley, Amadou T. Jallow, Jie Liu, Eric R Houpt, Andrew M. Prentice, Martin Antonio, Robin M Bernstein, Christopher L. Dupont, Brenda A. Kwambana-Adams

**Affiliations:** a Department of Clinical Sciences, Liverpool School of Tropical Medicine, Liverpool, L3 5QA, UK; b J. Craig Venture Institute, 4120 Capricorn Ln, La Jolla, CA 92037, USA; c Applied Sciences, Durban University of Technology, Durban, South Africa; d Medical Research Council Unit The Gambia at London School of Hygiene and Tropical Medicine, Fajara, Banjul, PO Box 273, The Gambia; e NIHR Global Health Research Unit on Mucosal Pathogens, Division of Infection and Immunity, University College London, London, United Kingdom; f Department of Health Data Science, University of Liverpool, Liverpool, UK; g School of Health and Life Sciences, Federation University; h Division of Infectious Diseases and International Health, Department of Medicine, University of Virginia, Charlottesville, Virginia, United States of America; i Department of Infection Biology, Faculty of Infectious and Tropical Diseases, London School of Hygiene and Tropical Medicine, London, UK; j Growth and Development Lab, Department of Anthropology, University of Colorado, Boulder, CO, United States of America

**Keywords:** Malnutrition, Gut microbiome, Enteric pathogens, West Africa, *Escherichia-Shigella*, Network analysis, Feature selection, Community detection

## Abstract

**Background:**

The specific roles that gut microbiota, known pathogens, and host energy-regulating hormones play in the pathogenesis of non-edematous severe acute malnutrition (marasmus SAM) and moderate acute malnutrition (MAM) during outpatient nutritional rehabilitation are yet to be explored.

**Methods:**

We applied an ensemble of sample-specific (intra- and inter-modality) association networks to gain deeper insights into the pathogenesis of acute malnutrition and its severity among children under 5 years of age in rural Gambia, where marasmus SAM is most prevalent.

**Findings:**

Children with marasmus SAM have distinct microbiome characteristics and biologically-relevant multimodal biomarkers not observed among children with moderate acute malnutrition. Marasmus SAM was characterized by lower microbial richness and biomass, significant enrichments in *Enterobacteriaceae,* altered interactions between specific *Enterobacteriaceae* and key energy regulating hormones and their receptors.

**Interpretation:**

Our findings suggest that marasmus SAM is characterized by the collapse of a complex system with nested interactions and key associations between the gut microbiome, enteric pathogens, and energy regulating hormones.  Further exploration of these systems will help inform innovative preventive and therapeutic interventions.

**Funding:**

The work was supported by the UK Medical *Research Council (MRC; MC-A760-5QX00) and the UK Department for International Development (DFID) under the MRC/DFID* Concordat agreement; Bill and Melinda Gates Foundation (OPP 1066932) and the National Institute of Medical Research (NIMR), UK. This network analysis was supported by NIH U54GH009824 [CLD] and NSF OCE-1558453 [CLD].

**Research in Context**

**Evidence before this study**

There is increasing evidence that the pathogenesis of acute malnutrition may be multifactorial and linked to microbial dysbiosis, the overgrowth of specific enteric pathogens in the gut and hormonal imbalance. However, most of this evidence has been generated from children with edematous severe acute malnutrition [SAM] in East Africa and Southeast Asia. However, despite malnutrition being prevalent in West Africa, little is known about the pathogenesis of severe and moderate acute malnutrition during childhood in this region. Unlike East Africa and Southeast Asian, non-edematous nutrition dominates in this region, making it a unique case study.

**Added value of this study**

Here we provide novel insights into the role of microbial dysbiosis, enteric pathogens, and host energy-regulating hormones in the pathogenesis of both severe and moderate non-edematous acute malnutrition among West African Children during outpatient nutritional rehabilitation. This study also demonstrates key differences in the microbiome structure and enteric infections between children with severe and moderate non-edematous acute malnutrition.

**Implications of all the available evidence**

As with edematous severe acute malnutrition, non-edematous SAM is also characterized by the collapse of a complex system including the gut microbiome, enteric pathogens, and energy regulating hormones, but in with different important features.  Future interventions should target these systems to improve the management and outcomes for children with acute malnutrition.

Alt-text: Unlabelled box

## 1 Introduction

Protein-energy malnutrition [PEM] is defined as a range of pathological conditions arising from inadequate calories and/or protein intake [1]. PEM in early childhood (< 5 years) is endemic in low-and middle-income countries and is a contributory factor in up to 50% of under 5 mortality [2]. Acute malnutrition or wasting is a manifestation of PEM and is defined by the World Health Organization as weight-for-height Z scores [WHZ] below the median of the WHO Growth Reference Standards by at least 2 standard deviations [3]. It has varying degrees of severity from moderate acute malnutrition [MAM] to severe acute malnutrition [SAM] which can present as: 1) kwashiorkor or edematous SAM; 2) marasmus or non-edematous SAM; and 3) marasmic-kwashiorkor [4]. The 2018 Gambia *Multiple Indicator Cluster Survey* estimated an acute malnutrition prevalence of 7.4% among children between 6-23 months of age [5], while the Gambia *National Micronutrient Survey* estimated an acute malnutrition prevalence of 5.8% for children under 59 months of age [6].

SAM has multifactorial causes [7], which may contribute to the poor performance of nutritional interventions in combating this public health challenge [8]. In addition to inadequate infant feeding practices and household food insecurity that are invariably critical factors, acute malnutrition may also develop, progress, or persist as a result of enteric pathogens [9] and gut mucosal barrier dysfunction [10,11] associated with unhygienic living conditions from early childhood. In addition, the pathology of acute malnutrition may vary across different populations giving rise to similar nutritional phenotypes. Research investigating how the gut microbiome plays a role in acute malnutrition have been mostly limited to kwashiorkor and based in countries in East and Southern Africa, South-East Asia, and Central America [12], [13], [14], [15] with substantial evidence that gut microbiome structure varies across regions [16,17]. While a previous metagenomic study has investigated acute malnutrition in the West African region [18], the focus was on the relationship between human milk oligosaccharide composition and microbiome composition.

The gut microbiome is complex and is only partially determined by the host genome. Several factors are involved in shaping the gut microbiome such as environmental modulation and diet [19,20]. For example, diets in low-and middle-income countries are often rich in complex plant polysaccharides compared to the energy-dense animal-derived foods and processed carbohydrates in high income countries ^7^. Differences in dietary carbohydrate composition select for bacteria able to metabolize the available nutrients and such differences have been observed when comparing the fecal microbiota between healthy children in sub-Saharan Africa and those in Europe [21,22]. Depending on diet, gastrointestinal microbes produce a plethora of metabolites that modulate the host's fitness, phenotype, and health [23]. Given the variation in gut microbiota between individuals and the interplay between microbial and host metabolites in relation to nutrition, it might be possible for nutritional interventions aimed at acutely malnourished children to be optimized by targeting them to the specific needs of each child or region.

To implement such a strategy, a comprehensive framework must be developed to characterize the interplay of endogenous and exogenous metabolism in the context of acute malnutrition. This complexity raises the question of how best to characterize the microbiome among children with SAM relative to well-nourished children. With a few exceptions, previous microbiome studies typically focus on individual components of the gut ecology (e.g. operational taxonomic units [OTU]) and describe microbiomes in terms of taxa abundance and diversity. Although these metrics are informative, they overlook the fundamental interactions between the microbial and host ecosystems. With the prospect of targeted probiotics and interventional therapeutics on the horizon, the introduction of a single or a few bacterial species may be insufficient for the long-term nutritional restoration of a dysbiotic ecosystem.

Microbiota-directed therapeutics are relatively new in the realm of medicine, but the fundamentals and conceptualization have been pioneered extensively by Gordon and colleagues [24], [25], [26], [27]. The hypothesis of undernutrition pathogenesis proposed in Blanton et al. 2016 is the following: 1) initial gut community is disrupted by one or more factors (e.g. enteropathogen competition); 2) gut dysbiosis provides opportunities for the virulence potential of pathobionts to be activated and enteropathogens to further establish themselves in the community and exacerbating dysbiosis; 3) impaired microbiome development impairs host immune response and defense; and 4) disruption of microbiota and host co-development induces the effects of undernutrition such as impaired muscle/bone growth, metabolism, and immune/gut barrier function.

As similar conditions of acute malnutrition can develop from different host-microbiome system configurations, the need for investigating the multifactorial nature of undernutrition and different mechanisms of pathogenesis is essential. The aim of our study was to investigate the interactions between the gut microbiome, enteric pathogens, and energy regulating hormones among rural Gambian children with non-edematous SAM (the predominant type of SAM in this region of The Gambia) during outpatient nutritional rehabilitation and MAM relative to well-nourished [WN] children. Our research leverages information gained from a sample-specific perspective towards providing insight into various mechanisms of pathogenesis and defining characteristics of the overarching nutritional status phenotypes. We applied innovative tools for analyzing these complex and dynamic interactions within an individual child.

## 2 Methods

### 2.1 Setting and context

The study was conducted in the East, West, and Central Kiang districts of rural Gambia. Rural Gambian infants are typically small at birth relative to international standards, show positive growth during the first few months of life, and then enter a period of profound growth faltering of both weight and length until 24 months of age [8]. Although breastfeeding up to 2 years of age is the norm, gastrointestinal infections are commonly acquired from as early as 3 months of age [28], [29], [30]. Most of the population are subsistence farmers and in recent years crop failure has been common, rendering them food insecure [31]. Housing conditions are often basic with animals living in close proximity to the humans. However, there has been increasing access to clean water and sanitation over the past 4 decades in some of the villages, although housing standards remain basic [32]. Most households rely on water from communal taps in the village and household level pit latrines for sanitation**.**

### 2.2 Study design and sample size

This was a cross-sectional observational sub-study of a quasi-experimental study that aimed to investigate the role of energy regulating hormones in the variable growth responses of rural Gambian children during nutritional rehabilitation. The baseline characteristics of the participants are published elsewhere [33]. In summary, children from 6 to 24 months of age who had presented to three rural primary health care services including the Medical Research Council Unit, The Gambia [MRCG] Keneba field station clinic, Soma Health Centre or villages covered by the Kwinella trekking team, from June 2013 to October 2014 were recruited. All participants recruited into the study underwent clinical assessments and assigned to one for the following groups: MAM, SAM, and well-nourished [WN (WHZ > -2)]; WN control participants were based on the anthropometric measurements ^3^. MAM is classified as WHZ between 2 and 3 standard deviations below the WHO growth reference standard or a mid-upper arm circumference [MUAC] between 115 and 125 mm while SAM is classified as WHZ less than 3 standard deviations below or MUAC < 115 mm or bilateral oedema [3]. SAM children were managed in an outpatient nutrition rehabilitation unit in rural Gambia with a service for ambulatory provision of intravenous antibiotics and limited capacity for overnight observation and management of children requiring intravenous fluids and nasogatric feeds for < 48 hours. Children were who HIV-infected or had significant medical complications requiring transfer secondary or tertiary level care were excluded.

All children with SAM and MAM received 28 days of ready to use therapeutic foods (RUTF) according to the WHO and Gambian guidelines for the integrated management of acute malnutrition [34]. All children with SAM received broad spectrum antibiotics including amoxycillin for 7 days according to international guidance for management of SAM. In addition, 11 (61%) controls and 9 (41%) children with MAM had antibiotics prescribed at recruitment. Pre-and 1-hour post prandial venous blood samples for analysis of energy-regulating hormones were collected from all children at recruitment and for children in the MAM and SAM groups at days 14 and 28. Stool samples were collected from all the children at recruitment and at follow up visits at days 14 and 28 for children that presented with MAM or SAM (tables 2,3). Terminology, abbreviations, and methodology sources are available in table 1.

**Table 1:** Terminology and abbreviations, Terminology and abbreviations for various concepts, data structures, and techniques used in this study.

Category	Name	Acronym	Description	Citation
**Data**	**Feature**		An individual measurable property or characteristic of a phenomenon being observed	
	**Operational taxonomic unit**	OTU	Groups of highly similar 16S rRNA sequences	
	**Modality**		One of the following datasets: (1) gut microbiome; (2) clinical measurements; and (3) pathogen markers	
	**Multimodal**		Data generated from multiple modalities or measurements	
**Phenotypes**	**Weight-for-Height Z-score**	WHZ	Compares a child's weight to the weight of a child of the same height and sex to classify nutritional status	[3]
	**Well-nourished**	WN	-2 < WHZ	[3]
	**Moderate acute malnourished**	MAM	-3 < WHZ ≤ -2	[3]
	**Severe acute malnourished**	SAM	WHZ ≤ -3	[3]
	**Undernourished**	UN	MAM or SAM. Used in the context aggregate networks and predictive modeling	
	**Protein energy malnutrition**	PEM	Protein-energy malnutrition defined as a range of pathological conditions arising from inadequate calories and/or protein intake.	[1]
**Modeling**	**Clairvoyance**		Feature selection algorithm leveraged for phenotype-discriminative community detection	[58]
	**Hierarchical Ensemble of Classifiers**	HEC	Graphical model where each internal node is a customized sub-model classifier with a unique feature set	[58]
	**Sub-model**		Machine-learning classification model used as internal node in a HEC model	
	**Leave subject out cross-validation**	LSOCV	Cross-validation designed to simulate performance on a new subject	This study
**Networks**	**Background network**	BN	Networks created from individuals who were WN for all	This study
	**Perturbed background network**	PBN	Networks created when adding in a query individual to the background network	This study
	**Sample-specific network**	SSN	Network with unique properties for each sample	[80]
	**Sample-specific perturbation network**	SSPN	Network created from perturbation between BN and SSPN distributions	This study
	**Aggregate network**	AN	Networks created from fitted sub-model coefficients	This study
	**Node**		The discrete objects within a network	
	**Edge**		Weighted connections between nodes	
	**Edge weight**		Association or perturbation strength of edge	
	**Perturbation**		Change in association strength of an edge between SSN and BN distributions	
	**Connectivity**	k	Sum of weighted edges connected to a note	
	**Scaled connectivity**	k∼	Scaled connectivity so total connectivity of all nodes sums to 1	
**Pathogenic** **E. coli**	**Enteropathogenic E. coli**	EPEC	EPEC E. coli are defined by the induction of a distinctive histopathology known as the attaching and effacing (A/E) lesion, which is characterized by the effacement of the intestinal microvilli and the intimate attachment of the bacteria to the host epithelial surface	[81,82]
	**Enteroaggressive E. coli**	EAEC	EAEC E. coli is defined as a diarrheal pathogen based on its characteristic aggregative adherence (AA) to HEp-2 cells in culture and its biofilm formation on the intestinal mucosa with a “stacked-brick” adherence phenotype.	[83]
	**Enterotoxigenic E. coli**	ETEC	ETEC E. coli are a pathogenic variant or pathovar of E. coli defined by production of diarrheagenic heat-labile (LT) and heat-stable (ST) enterotoxins.	[84]

### 2.3 Ethics approval

Ethical approval to conduct this study was granted by the *Joint Medical Research Council Unit The Gambia* and *Gambia Government Ethics Committee*, L2015.14. All children were recruited into the study with written informed parental consent.

### 2.4 Clinical measurements for energy regulating hormones and receptors

The hormones were measured in plasma and blood as we described previously [33]. We calculated molar excess soluble leptin receptor [sOB-R] concentrations divided by leptin concentrations multiplied by 0.13 (i.e. $\frac{\left[sOB-R\right]}{\left[Leptin\right]}\times \;0.13$), referred to as *molar* (table 4), a formula following Stein et al. 2006 in their study that sought to elucidate the role of the sOB-R and its regulation in children with protein energy malnutrition during nutritional recovery [35].

### 2.5 DNA extraction and enteric pathogen detection

Stool specimens underwent pre-treatment including glass bead beating followed by nucleic acid extraction using the QIAamp® Fast DNA stool mini Kit (Qiagen, Manchester UK). Efficiency of the nucleic acid extraction and amplification was monitored through external controls, bacteriophage MS2 and phocine herpesvirus as previously described[36]. Detection of 19 enteric pathogens (bacteria, viruses, helminths and protozoa) was performed using a custom TaqMan Array Card [TAC] previously developed to investigate the etiology of moderate-to-severe diarrhea among children[36]. TAC assays were run on the QuantStudio™ 7 Pro Real-Time PCR System (ThermoFisher Scientific, Loughborough, UK). The detailed procedures for setting up the TAC and cycling condition have been described elsewhere [36]. The gene target for *Escherichia/Shigella* in the custom Enteric TAC is the invasion plasmid antigen H (*ipaH)* which is carried by *Shigella* species and enteroinvasive *E. coli* (EIEC) [36].

Detections were considered negative if the cycle thresholds [ct] were greater than 35. Results were validated and discarded if any of the controls failed. The following nested pathogen markers were merged to avoid interdependence of variables: (1) *adenovirus* (*adenovirus*_*f, adenovirus_pan*); (2) *cryptosporidium* (*cryptosporidium, cryptosporidium*_*hominis, cryptosporidium*_*parvum*); (3) *giardia* (*giardia, giardia*_*a, giardia_b*), *norovirus* (*norovirusgi, norovirusgii*); and (4) *stec* (*stec_stx1, stec_stx2*). Pathogens that were detected in fewer than 3 samples were removed prior to downstream analysis including: *rotavirus_g1, vcholerae, cdifficile, cyclospora*, and *aeromonas*. As *epec_bfpa* and *epec_eae* are different gene targets on the *E. coli* adherence factor (EAF) plasmid and EPEC chromosome [37], respectively, we kept these separate to account for potential detection bias and mixed EPEC communities within a sample.

## 3 Quantification and statistical analysis

### 3.1 16S rRNA, sequencing and operational taxonomic units

16S ribosomal RNA [rRNA] gene analysis was used to investigate the bacterial component of the gut microbiome. The 16S rRNA gene libraries (regions V3 and V4) were prepared using the Nextera® XT Index Kit (Illumina, Essex, UK) followed by multiplexed sequencing using the MiSeq Reagent Kit on the Illumina MiSeq System (Illumina, Essex, UK) following the manufacturer's protocol. Extraction and PCR controls were also included to detect possible contaminants. Operational taxonomic units [OTU] were generated *de novo* from Illumina sequence reads using *UPARSE* [38] and *mothur* [39] open-source bioinformatics tools. Paired-end reads were trimmed of adaptor sequences, barcodes, and primers prior to assembly, followed by discarding low quality reads and singletons. After a de-replication step and abundance determination, sequences were filtered for chimeras and clustered into OTUs. *UPARSE* has a built-in filter for chimera detection and removal, *UCHIME2* (http://www.biorxiv. org/content/early/2016/09/09/074252), which uses the highly curated SILVA database. To predict taxonomy, we used the Wang classifier, and bootstrapped using 100 iterations. We set *mothur* to report full taxonomies only for sequences where 80 or more of the 100 iterations were identical (cutoff = 80). Taxonomies were assigned to the OTUs with *mothur* using version SSU Ref NR 99 123 of the SILVA 16S ribosomal RNA database as the reference. Tables with OTUs and the corresponding taxonomy assignments were generated and used in subsequent analyses with annotations detailed in table S2.

### 3.2 Alpha and beta diversity for microbial composition

**Figure 1:**
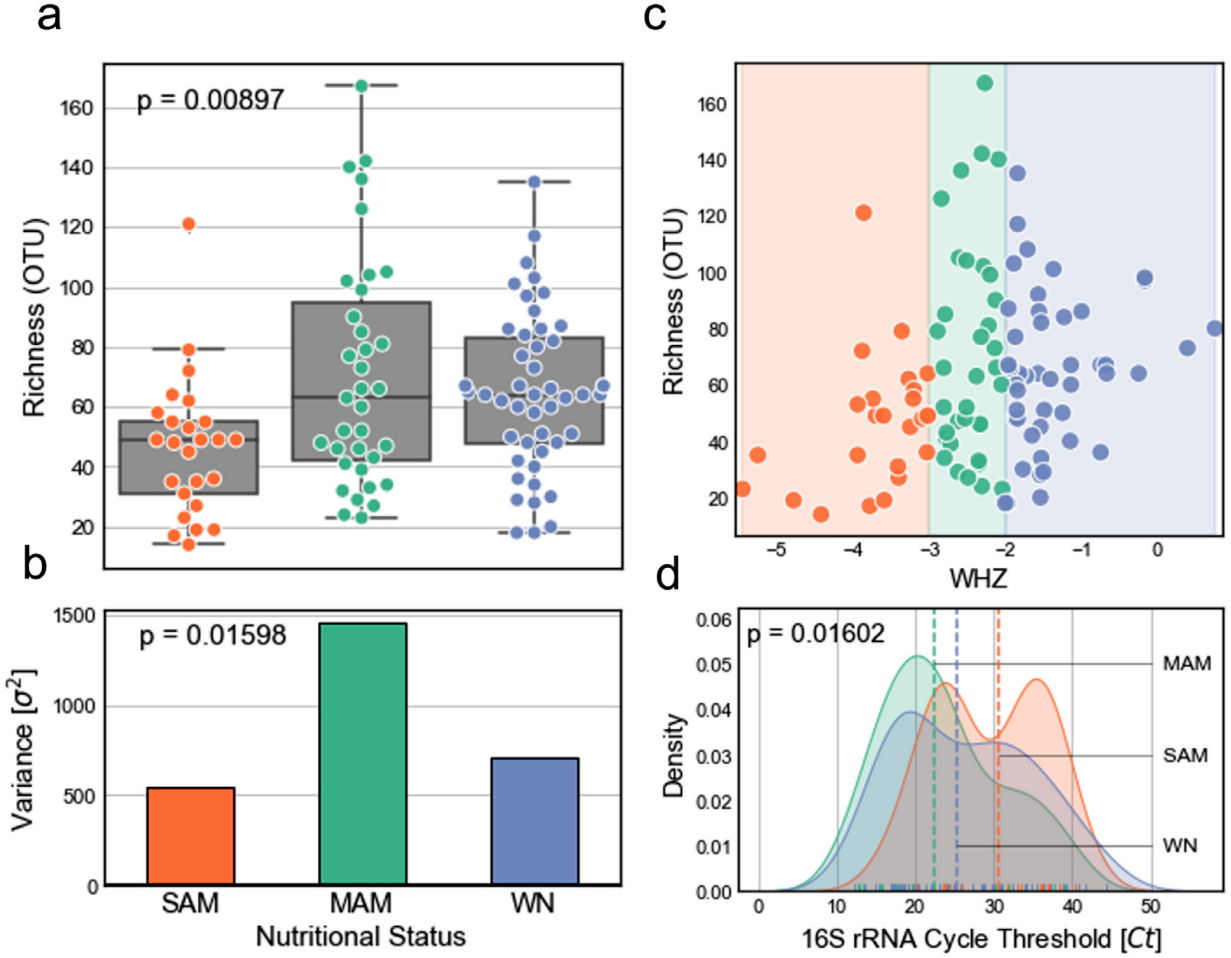
Microbial alpha diversity in the context of WHZ classifications, (a) Box-plot showing number of detected OTUs (richness) for each phenotype with inner values as interquartile range (Kruskal-Wallis H-test, p = 0.024). (b) Variance of microbial richness for each phenotype (Levene test, p = 0.019). (c) Microbial richness with respect to sample WHZ scores. (d) Cycle thresholds for 16S rRNA qPCR (Kruskal-Wallis H-test, p = 0.0169).

Our preferred alpha diversity metric is microbial richness which measures the number of unique components detected within a particular sample. We measured alpha diversity for various levels including Family, Genus, Species, and OTU (figure 1 and supplementary figure 2). For all downstream OTU analysis, we removed OTUs that were not observed in at least 12% of the samples resulting in 155 prevalent OTUs. As OTU counts are compositional, we used a compositionally valid approach with the following protocol: (1) inferring a phylogenetic tree from OTU centroids using *FastTree v2.1.10* [40] with default parameters; (2) transforming abundances with the isometric log ratio transform using phylogenetic tree as the basis [41], [42], [43]; and (3) computing pairwise Euclidean distance [44].

### 3.3 Predictive functional profiling of microbial communities

We used *PICRUSt2 v2.3.0_b* to predict functional profiles for each sample [45] as human microbiomes are modeled with high performance [46]. We tested for differential enzyme abundance for MAM and SAM nutritional status phenotypes, with WN as reference, using the Mann-Whitney U test followed by Benjamini-Hochberg multiple hypothesis testing for adjusted p-values; statistical significance threshold set at 0.05. Statistical tests were run only for predicted enzymes that had more than 20 non-zero abundances in both WN and MAM/SAM classes as suggested by *SciPy* documentation. We used the GSEA's *Prerank* module (via *GSEApy v0.9.8*) to assess if any KEGG pathways were enriched in differentially abundant enzyme sets using 1 – adjusted p-values as our pre-ranked enzyme weight [47]. For *Prerank,* we used FDR < 0.25 for statistical significance as recommended by GSEA authors.

### 3.4 Modality-specific differential abundance analysis

We calculated differential abundance for each modality separately using appropriate methods for each data type. For our compositional microbiome data, we calculated differential abundance between WN/MAM and WN/SAM with the *ALDEx2 v1.20.0* R package using a Wilcoxon signed-rank test with Benjamini-Hochberg corrected p-values [48]. Our clinical measurements were non-integer continuous data and we calculated differential abundance using a Kruskal-Wallis H-test with normalized abundances [44]. Our pathogenic screening data was binary, so we used Fisher's exact test with a contingency table populated from the number of detected events and non-detected events for each nutritional status category. All p-values were corrected for multiple tests using Benjamini-Hochberg adjustment with an adjusted p-value threshold of 0.05 for statistical significance.

### 3.5 Linear mixed-effects regression

We used mixed linear-effects models to regress out variation from uncontrollable variables associated with the participant. In particular, we used the following regression model: *Feature_j_* ∼ FixedEffects(*Age* + *Height* + *Sex*) + RandomEffect(*Participant Identifier*) where *Feature_j_* represents a normalized/transformed feature vector. *Age* and *Height* were z-score normalized prior to modeling. Features that statistically covary with microbial abundance, clinical measurements, and enteric pathogen presence are available in supplementary figure 4. We adjusted for these fixed and random effects in all subsequent analyses. We implemented multiple linear regression models using the *MixedEffectsLinearModel* object in *Soothsayer* [49] with the *statsmodels* [50] backend.

### 3.6 Network structure and visualization

Networks are graphical structures used to represent relationships between discrete objects where these discrete objects are referred to as nodes and connections between nodes as edges [51]. The connections are weighted by a numeric value.

The nodes represent individual features from the following modalities: (1) fecal gut microbiome; (2) clinical measurements; and (3) pathogenicity markers, while the edges between nodes represent intra- and inter-modality associations. To prepare the modalities for multimodal pairwise associations, we scaled the clinical measurements using z-score normalization and center log-ratio transformation for the fecal gut microbiome. As our multimodal data were only partially compositional, including an addition of binary and non-integer continuous data, we were not able to use proportionality metrics [52,53] and implemented a bootstrapped Pearson's correlation as our association metric.

Traditional ball-and-stick networks were visualized using the *NetworkX* Python package [54]. Hive plots were implemented using the *HiveNetworkX* Python package [55,56]. Edge and node connectivity RainCloud plots were generated using the *PtitPrince* Python package [57].

### 3.7 Sample-specific perturbation networks

Our objective with a sample-specific approach was to maximize the available data and quantify the amount by which a particular sample perturbs a biologically-relevant background distribution; we refer to this as a sample-specific perturbation network [SSPN]. We define a perturbation in the context of SSPNs as a change in association strength of an edge between background network [BN] and perturbed background network [PBN] distributions (supplementary figure 5). For the background cohort, we used data from participants whose nutritional status was WN for all visits (n=25, table 3), which allowed us to calculate SSPNs for participants that deviated to or from WN between visits (n=82 samples, table 2).

**Table 2:** Baseline characteristics, Baseline characteristics of children with respect to nutritional status as defined in a previous study [33].

	Nutritional category			P
	WN (N=22)	MAM (N=18)	SAM (N=20)	
Age in months, median (IQR)	12.75 (10.2, 19.3)	16.5 (12.0, 22.0)	12.0 (10.3, 16.5)	0.22^a^
Age of weaning in months, median (IQR)	6.0 (5.0, 6.0)	6.0 (6.0, 6.0)	6.0 (5.5, 6.0)	0.82^a^
WHZ, median (IQR)	-1.2 (-1.8, 0.1)	-2.6 (-2.8, -2.1)	-3.4 (-3.9, -3.2)	
WAZ, median (IQR)	-1.5 (-1.7, -0.1)	-2.8 (-3.1, -2.1)	-3.2 (-3.4, -2.9)	<0.001^a^
HAZ, median (IQR)	-0.7 (-1.8, 0.03)	-1.7 (-2.5, -1.0)	-1.9 (-2.3, -0.9)	0.08^a^
Salivary CRP, ng/mL, median (IQR)	2.9 (2.4, 4.1)	4.9 (2.8, 10.3)	5.6 (4.1, 9.9)	0.04^a^
Urinary tract infections, n (%)	4 (19)	2 (12)	1 (6)	0.47^b^
* Diarrhea, n (%) **Antibiotics prescribed	2 (11) 9 (41)	4 (29) 11 (61)	11 (58) 18 (90)	0.01^b^ 0.003^b^
Females, n (%)	11 (50)	8 (44)	10 (50)	0.90^b^

Abbreviations: IQR Interquartile range, MAM Moderate Acute Malnutrition; SAM Severe Acute Malnutrition; WHZ weight-for-height z-score; WAZ weight-for-age z-score; HAZ, Height-for-age z-score.

a Kruskal-Wallis test.

b Fisher's exact test.

⁎ Diarrhea defined according to the WHO guidance of passage of 3 or more loose or liquid stools per day or more frequent passage than normal for the child.

⁎⁎ If the child had any antibiotics prescribed during the study (irrespective of type, mode of administration or duration).

More specifically, we created SSPNs for 82 samples using 1000 sub-sampled permutations and a sampling size [***n***] of 15 with each draw denoted as ***k***. The ***BN*** distribution was created by pseudo-randomly (seed=0) drawing ***n*** samples from the background cohort without repetition, creating a pairwise multimodal association network, and stacking the edges for all permutations allowing us to calculate a distribution of background edge weights; ***BN*** is parameterized by a matrix of ***k*** draws and ***m*** edges. For each query sample ***i***, we repeated the ***BN*** creation process using the same sub-sampled sample draws but we added the query sample to build a distribution of edges for our query PBN distribution [***PBN_i_***]. For each edge ***j****,* we calculate the difference in means for the query network distribution ***PBN_ij_*** and background network distribution ***BN_j_*** to build ***SSPN_i_***(a vector for sample ***i*** with ***m***)***_._*** Each edge ***j*** in ***SSPN_i_*** is a measure of how much the addition of query sample ***i*** perturbs the background system. Although the BN and PBN distributions are intermediate data structures used solely to calculate SSPNs, these network distributions are fundamental to capturing true variability within a study cohort while also being robust to sample outliers. More algorithm details are available in the source code mentioned below.

The range of values for each SSPN edge weight is in the closed interval [-2,2] where positive and negative values indicate an increase and decrease in association strength, respectively. Each SSPN was stacked to yield a perturbation matrix [***M***] of 82 samples and 14,270 edges, where each row ***i*** corresponds with a sample, each column ***j*** represents an edge, and each ***M_i,j_*** indicates a perturbation value*.* This matrix structure allowed us to efficiently store networks and leverage machine-learning methods that require 2-dimensional data. These methods are open-sourced in our *EnsembleNetworkX* Python package using the *SampleSpecificPerturbationNetwork* object.

### 3.8 Phenotype-discriminative network community detection

We decomposed our classification of nutritional status phenotypes into step-wise binary classifications of severity using highly interpretable algorithms for each step. Each step in our hierarchical classification scheme can be interpreted which maximizes the available information content.

For our training data, we use the perturbation matrix ***M****,* containing our SSPNs, as our feature matrix and their associated nutritional status classifications as the target vector. To ensure our associations were not biased, and remained clinically relevant, we used a stringent Leave Subject Out Cross-Validation [LSOCV] to simulate classification performance on new participants and LSOCV accuracy as a proxy for reliability in clinical relevance. Although our primary objective was not to classify sample nutritional status based on SSPNs, we used LSOCV to simulate classification performance on new participants and LSOCV accuracy as a proxy for the community detection capabilities of our feature selection analysis.

In particular, we implemented a Hierarchical Ensemble of Classifiers [HEC] model to partition a single tertiary classification into two step-wise binary classifications. Our HEC model asks the following questions: (1) is the participant at this visit WN or acutely malnourished and (2) if undernourished, is the participant at this visit MAM or SAM? We translate these human interpretable questions directly to machine interpretable tasks by building a step-wise classification algorithm that trains each decision using a subset of the samples and a subset of features. In particular, we produce the following HEC model: 1) the first binary classification performed by sub-model *y1* discriminates between WN and MAM/SAM, collectively referred to as undernourished [UN] class for here forth; and (2) the second binary classification performed by sub-model *y2* differentiates between MAM and SAM. The order of each binary class mentioned prior corresponds with a 0 and 1 in a standard logistic regression classification. In these fitted logistic regression models, a positive coefficient corresponds with an increase in likelihood that a sample is classified as the 2^nd^ class (i.e. UN in sub-model y1 and SAM in sub-model y2) and vice versa. Despite being the logical progression when diagnosing severity, these human interpretable questions that define the step-wise decisions in our HEC model were data-driven and determined by *Soothsayer's Topology* method using only the training data*.*

These sub-models were optimized using the *Clairvoyance* feature selection algorithm (available within the *Soothsayer* package) which returns a set of features, edges in this case, and sub-model hyperparameters that result in the corresponding LSOCV accuracy ^49,58^. We used feature selection with our SSPNs to identify edges that are associated with discriminating phenotypes (i.e. paths within the graph). The output of the feature selection algorithm includes 3 main elements including the following: (A) hyperparameters for the sub-model classifier; (B) the edge set used during model fitting; and (C) the LSOCV accuracy using a combination of using (A) hyperparameters with (C) features. We selected the set of edges and hyperparameters with the highest LSOCV accuracy to build classification sub-models as internal nodes in the HEC model using *Soothsayer's HierarchicalClassifier* method. Edge sets ***Edges_y1_*** and ***Edgess_y2_*** were used to build sub-models *y1* and *y2,* respectively, representing the smallest subset of features that most effectively discriminate between nutritional status phenotypes. *Clairvoyance* feature selection and LSOCV accuracy have been adapted from Espinoza and Dupont et al. 2021 which were developed to model antimicrobial mechanism-of-action [58].

We used L2-regularized logistic regression classifiers for both sub-models ***y1*** and ***y2*** with inverse regularization strength of 1.0 and 0.106, respectively, as these machine-learning algorithms often perform with high accuracy and are human interpretable compared to “black-box” algorithms such as neural networks [59]. Logistic regression classification models are easily interpreted as each feature has a coefficient and the magnitude of these coefficients reflects the influence a particular feature has on the classification.

We used *Clairvoyance* with the following parameters: *–model_type logistic,tree –n_iter 500 –min_threshold None, 0.0, 0.1, 0.2, 0.3, 0.4, 0.5, 0.6, 0.7, 0.8, 0.9 –percentiles 0.0, 0.1, 0.2, 0.3, 0.4, 0.5, 0.6, 0.7, 0.8, 0.9, 0.91, 0.92, 0.93, 0.94, 0.95, 0.96, 0.97, 0.98, 0.99 –method bruteforce –early_stopping 100 –cv LSOCV.tsv* where LSOCV.tsv contains custom training and testing pairs organized by participant.

### 3.9 Aggregate networks

We developed ANs as a method to quantify the predictive capacity of a node, edge, or subgraph in the context of discriminating phenotypes. ANs serve as an edge framework for building phenotype-discriminative SSPNs whose weights are populated by sample-specific perturbations from ***M***. In the case of this study, AN edge weights represent L2-regularized logistic regression coefficients; though, it would be seamless to incorporate L1-regularization or feature importance metrics from tree-based algorithms. More specifically, ***Edges_y1_*** and ***Edges_y2_*** were used to build ***AN_y1_***and ***AN_y2_***with edges weighted by the fitted ***y1*** and ***y2*** sub-model parameters. A positive coefficient in sub-model ***y1*** and ***y2*** corresponds with an increased likelihood for classifying a sample as UN or SAM, respectively, where UN represents either MAM or SAM. The inverse of this is true as well where a negative coefficient represents a decrease in likelihood in said classes. Aggregate and sample-specific perturbation networks were implemented using *Graph* objects in *NetworkX* [54] and *EnsembleNetworkX*.

### 3.10 Connectivity in the context of sample-specific networks and aggregate networks

Network connectivity [*k*] is a metric used to quantify the influence a particular edge, node, or group of nodes has within a system. In this study, we implemented weighted-degree as our connectivity metric which can be measured at different levels. We define connectivity at the edge level to represent the edge weight of a pair of nodes, while at the node level connectivity refers to the sum of weighted edges connected to a node. These connectivities can be grouped such as quantifying the total connectivity of a subset of edges (as in the case for node connectivity by grouping edges connected to a node), a subset of nodes, or the entire network itself. Scaled connectivity [*k^∼^*] normalizes the node connectivity values so the total connectivity within a network sum to 1 and can be used to compare networks with different numbers of nodes or edges; such is the case for comparing ANs.

Network connectivity is interpreted differently depending on the network context. In the context of SSPNs, edge weight represents perturbation magnitude; that is, the change in association of a query PBN distribution with respect to a BN distribution. Therefore, the total connectivity within a SSPN measures how much a particular sample perturbs the background associations with respect to the edges in the network. In the context of ANs, L2-regularized logistic regression coefficients are used as edge weights and can be interpreted as the influence of an edge in predicting nutritional status phenotypes. Therefore, the total connectivity within ANs is the combined influence of the edges in their predictive capacity.

### 3.11 Recovery scores

Our dataset contains time-ordered samples for many of the participants. We developed an edge recovery score [***r***] to quantify the amount in which an edge contributes to weight recovery; more specifically, the transition from UN to WN. As SSPN edge weight indicates a perturbation between the PBN and BN distributions, we identified perturbations relevant to nutritional status recovery by selecting for edges in consecutive time-ordered SSPNs that have the following properties: (1) greatest change in perturbation of associations between visits *t_n_* and *t_n + 1_*; and (2) smallest perturbation magnitude from the BN distribution at *t_n + 1_* where *t_n_* and *t_n + 1_* phenotypes represent UN and WN, respectively.

For each participant that recovers their nutritional status, we calculate ***r*** using the following equation: $r_{i,j}\left(x,y\right)=\;\frac{{\left|y-x\right|}^{2}}{4\;+\;6|y|}$ where x and y represent edge weight *j* for participant *i* at visits *t_n_* and *t_n + 1_*, respectively. The expression ${\left|y-x\right|}^{2}$ corresponds with property (1) while 4+6|y| corresponds with property (2). This equation bounds ***r*** within the closed interval [0,1] where higher values correspond to larger potential influence in weight recovery (figure 6a). Non-zero recovery scores were grouped by (1) MAM → WN and (2) SAM → WN transitions between *t_n_* and *t_n + 1_.* These distributions were plotted using RainCloud plots and outlier thresholds were determined using the 1.5*IQR + Q3 between the distributions where IQR is interquartile range and Q3 is the 75^th^ percentile. The outlier thresholds for MAM → WN (0.449 *r*) and SAM → WN (0.452 *r*) recovery scores were very similar so we used the minimum value of 0.449 *r* as our consensus threshold for outliers (figure 6b).

The recovery score metric condensed the information content of complex multimodal time-ordered SSPNs into a single human interpretable metric. We designed the recovery score to demonstrate the following properties for each edge: (1) a large difference between an undernourished visit and a consecutive WN visit; and (2) a small edge weight for WN. Emphasizing these properties allowed us to collapse the temporal dimension, the sign of edge weights, and focus specifically on edges specifically to the recovery of an individual participant.

### 3.12 Role of funding source

The funding sources had no role in the design of this study and did not have any role in the study design, data collection, data analyses, interpretation, or writing of report. or decision to submit results.

## 4 Results

### 4.1 Dataset overview

Sixty children were recruited to the study (20 SAM, 18 MAM, 22 WN) with visit-specific samples detailed in tables 2,3. All the children with SAM presented with non-edematous SAM. The median age of the children was 12.0 months for SAM, 16.5 months for MAM, and 12.75 months for WN at recruitment. A total of 54 children had stool specimens available in this study that passed quality control; 25, 35 and 47 stool samples were collected from the children in SAM, MAM and WN groups based on their nutritional status at baseline. Stool samples were collected only at baseline among the WN children. For the children in MAM and SAM, stool samples were collected at baseline, Day 14 and Day 28 (table 3 and supplementary figure 2e).

**Table 3:** Subjects and samples with respect to nutritional status, The number of subjects and samples with respect to nutritional status and status changes.

Visits	Category	Phenotype	N Participants	N Samples
**All visits**	**Nutritional status**	SAM	16	25
		MAM	25	35
		WN	34	47
		Total	75 (54 unique)	107
	**Maintained nutritional status**	SAM	4	10
		MAM	10	19
		WN	20	25
**Follow-up visits**	**Nutritional status**	SAM	15	24
		MAM	23	33
		WN	19	32
		Total	57 (36 unique)	89
	**Maintained nutritional status**	SAM	3	9
		MAM	8	17
		WN	5	10
	**Recovery**	t=0 → t=14		
		SAM → MAM	3	-
		SAM → WN	5	-
		MAM → WN	5	-
		Total	13	
		t=14 → t=28		
		SAM → MAM	2	-
		SAM → WN	0	-
		MAM → WN	1	-
		Total	3	-
	**Decline**	t=0 → t=14		
		WN → MAM	0	-
		WN → SAM	0	-
		MAM → SAM	0	-
		Total	0	-
		t=14 → t=28		
		WN → MAM	3	-
		WN → SAM	0	-
		MAM → SAM	1	-
		Total	4	-

Following nutritional intervention, 36 participants with acute malnutrition (i.e. MAM and SAM) were included in follow up visits. Of these follow up visits, 16 participants maintained a consistent nutritional status throughout all visits (SAM: 3 participants, MAM: 8 participants, and WN: 5 participants). Sixteen participants showed signs of recovery of at least one nutritional status level (i.e. SAM → MAM, SAM → WN, or MAM → WN), with 13 participants recovering between *t_0_* → *t_14_* and 3 participants between *t_14_* → *t_28_* visits (supplementary figure 2e). Four participants showed signs of decline of at least one nutritional status level (i.e. WN → MAM, WN → SAM, or MAM → SAM) between *t_14_* → *t_28_* visits. Multiple modalities were measured including: 1) fecal microbiome 16S rRNA sequencing (388 OTUs); 2) 12 clinical measurements related to immune activation, inflammation, and energy regulating hormones, and 3) qPCR derived presence of 23 viral, fungal, protozoan and bacterial enteric pathogens.

### 4.2 Fecal gut microbial composition and functional profiling predictions

There were a total of 388 high quality OTUs that passed quality control. The number of 16S ribosomal (r)RNA gene reads mapped to OTUs ranged from 392 - 92,910 and the number of detected OTUs (richness) ranged from 14 - 167 per sample (figure 1 and supplementary figures 1,2a-d). The microbial richness of SAM samples was statistically lower than both WN and MAM samples (figure 1a,c; Kruskal-Wallis H-test, p = 0.009). MAM samples had statistically higher variance compared to WN and SAM (figure 1b; Levene test, *p* = 0.016). The variance in richness was the highest for WHZ in the range (-3,-1), the entirety of MAM samples and the lower WHZ of WN samples (figure 1c).

**Figure 2:**
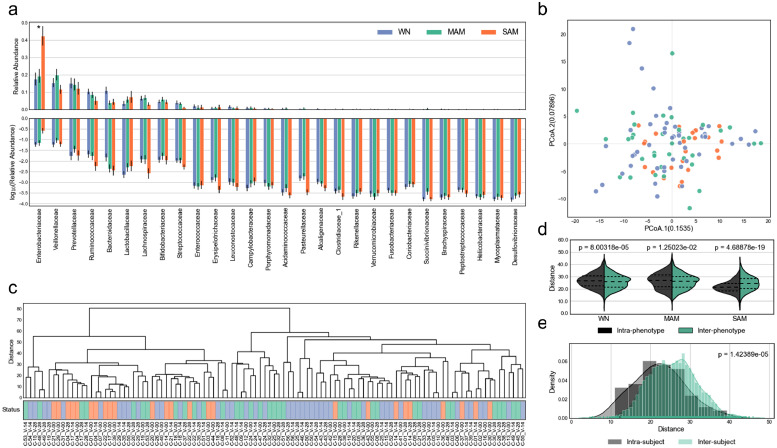
Taxonomic abundance and microbial beta diversity, (a) Relative abundance of OTUs summed by family-level taxonomy displayed using (A_Top_) linear and (A_Bottom_) log scales (Kruskal-Wallis H-test, adjusted p = 0.0246). (b) Principal coordinates analysis plot using phylogenetic isometric log-ratio transformation [PHILR] and Euclidean distance as precomputed distance matrix. (c) Hierarchical clustering using PHILR distance matrix (d,e) Intra- and inter-grouping diversity using PHILR distance matrix for (d) phenotype (Mann-Whitney rank test) and (e) participant (Mann-Whitney rank test, p = 1.424e-5). Error bars represent standard error of the mean.

We observed evidence of differences in 16S rRNA qPCR *Ct* values between nutritional status phenotypes (figure 1d; Kruskal-Wallis H-test, *p* = 0.016). MAM samples exhibited the lowest median *Ct* and thus the highest relative biomass on average (22.5 *Ct*), while SAM exhibited the lowest (30.5 *Ct*) with WN in between (25.4 *Ct*). A bimodal distribution of SAM *Ct* values with the lower peak at ∼24 *Ct* and the upper at ∼35 *Ct* suggested that while SAM samples have similar community composition (figure 2), in a substantial number of cases the bacterial community had collapsed from a numerical perspective.

The relative abundances of bacterial phyla were very similar across all nutritional status phenotypes with a few notable exceptions (figure 2a). The combination of *Firmicutes* (WN = 46%, MAM = 50%, SAM = 33%), Bacteroidetes (WN = 28%, MAM = 20%, SAM = 17%), and Proteobacteria (WN = 20%, MAM = 23%, SAM = 45%) constitute more than 90% of the microbial abundance regardless of the participant's nutritional status with respect to visit. We did not observe any components at any level of taxonomy that were differentially abundant among children with MAM relative to WN. We did however observe an enrichment in *Enterobacteriaceae* abundance (Wilcoxon signed-rank test, adjusted p = 0.018) for SAM (µ = 42%) relative to MAM (µ = 19%) and WN (µ = 18). The only differentially abundant OTU was an unclassified *Klebsiella* (*Otu000014*) with an enrichment in SAM (µ = 16%) relative to MAM (µ = 6%) and WN (µ = 3%) (adjusted p = 0.0442).

*Beta* diversity analyses did not reveal any defining global patterns associated with WN, MAM, or SAM (figure 2b,c). Despite this lack of qualitative separation in ordination space and through hierarchical clustering, we found evidence of differences between intra-and inter- nutritional category beta diversity (figure 2d; Mann-Whitney rank test, p < 0.001). The beta diversity of microbial communities from the same participants was lower than within a nutritional category (figure 2e; p < 0.001), despite some participants transitioning across nutritional categories during follow-up.

Functional content predictions of the gut microbiome were performed to gain insight into potential metabolic characteristics of each nutritional status phenotype. Predictive functional profiles produced 1390 predicted enzymes that were inferred from the fecal microbiome There were not any differentially abundant predicted enzymes relative to WN in MAM but 81 enzymes were differentially abundant in SAM. This set of 81 enzymes were enriched in 4 KEGG pathways including *map01120* (Microbial metabolism in diverse environments), *map00360* (Phenylalanine metabolism), *map00362* (Benzoate degradation), and *map01100* (Metabolic pathways).

### 4.3 Clinical measurements related to energy-regulating hormone

**Figure 3:**
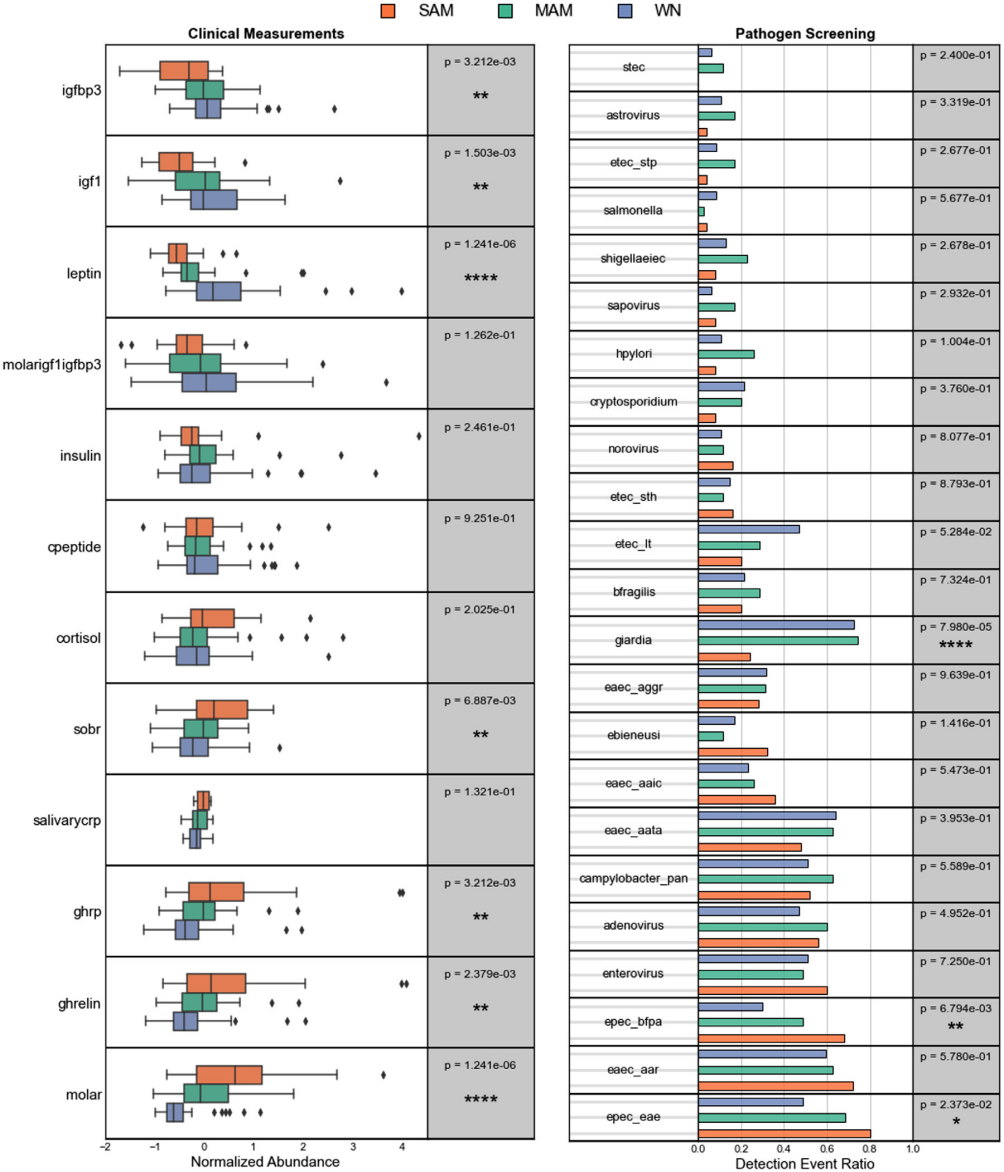
– Clinical measurement and pathogen abundance, (a) Boxplots showing distributions of normalized clinical measurements with respect to nutritional status with whiskers indicating IQR. Statistical test used was Kruskal-Wallis H-test. (b) Ratios of positive pathogen detection events with respect to nutritional status. Statistical test used was Fisher's exact test.

We explored the differences in plasma levels of key child growth and energy-regulating hormones at baseline for all the children and at days 14 and 28 for children with MAM and SAM. The annotations and descriptions for clinical measurements are summarized in table 4. Several clinical measurements were differentially abundant between nutritional status categories. In particular, *leptin* (Kruskal-Wallis H-test, adjusted p < 0.001), IGF-1 [*igf1*] (adjusted p < 0.002), and IGF-binding protein-3 [*igfbp3*] (p = 0.003) increased with nutritional status in that WN have the highest levels (figure 3a). As these statistics are associated with nutritional status which are dependent on WHZ, correlations between normalized clinical measurements and WHZ scores showed that *leptin* (Pearson p < 0.001), IGF-1 (p < 0.003) and its binding protein IGFBP-3 (p < 0.001) were indeed positively correlated with WHZ. In contrast, the molar excess of soluble leptin receptor (sOBR)/leptin [*molar*] (adjusted p < 0.001), *ghrelin* (adjusted p = 0.002), ghrelin receptor [*ghrp*] (adjusted p = 0.003), and sOB-R [*sobr*] (adjusted p < 0.007) measurements decreased monotonically with nutritional status in that SAM have the highest levels (figure 3a). As expected, the molar excess of sOBR/leptin (p < 0.001), *ghrelin* (p = 0.012), *ghrp* (p = 0.012), and *sobr* (p = 0.011) levels were inversely correlated with WHZ.

**Table 4:** Clinical measurements and pathogenic markers, Description of clinical measurements and pathogenic markers used in study with respect to shortened feature label.

Feature	Modality	Description
**sobr**	clinical	Fasting soluble leptin receptor [sOB-R] concentration
**leptin**	clinical	Fasting leptin concentration
**ghrp**	clinical	Fasting ghrelin receptor [ghrelinR] concentration
**ghrelin**	clinical	Fasting ghrelin concentration
**molar**	clinical	Fasting molar excess sOB-R concentration/leptin ratio
**igf1**	clinical	Fasting insulin-like growth factor 1 [IGF-1] concentration
**igfbp3**	clinical	Fasting insulin-like growth factor binding protein 3 [IGFBP-3] concentration
**molarigf1igfbp3**	clinical	Fasting molar excess of IGF-1 concentration/IGFBP-3 ratio
**insulin**	clinical	Insulin concentration
**salivarycrp**	clinical	Salivary C-reactive protein concentration
**cortisol**	clinical	Cortisol concentration
**astrovirus**	pathogen	Astrovirus
**bfragilis**	pathogen	Bacteroides fragilis
**campylobacter_pan**	pathogen	Campylobacter pan-genomes
**cryptosporidium**	pathogen	Cryptosporidium
**eaec_aaic**	pathogen	Enteroaggregative Escherichia coli aaiC gene
**eaec_aar**	pathogen	Enteroaggregative Escherichia coli Aar gene
**eaec_aata**	pathogen	Enteroaggregative Escherichia coli aatA gene
**eaec_aggr**	pathogen	Enteroaggregative Escherichia coli AggR gene
**ebieneusi**	pathogen	Enterocytozoon bieneusi
**enterovirus**	pathogen	Enterovirus
**epec_bfpa**	pathogen	Enteropathogenic Escherichia coli bfpA gene
**epec_eae**	pathogen	Enteropathogenic Escherichia coli eae gene
**etec_lt**	pathogen	Enterotoxigenic Escherichia coli heat-labile enterotoxin gene
**etec_sth**	pathogen	Enterotoxigenic Escherichia coli heat-stable enterotoxin gene
**etec_stp**	pathogen	Enterotoxigenic Escherichia coli heat-stable enterotoxin gene
**giardia**	pathogen	Giardia duodenalis
**hpylori**	pathogen	Helicobacter pylori
**salmonella**	pathogen	Salmonella
**sapovirus**	pathogen	Sapovirus
**shigellaeiec**	pathogen	Shigella/Enteroinvasive Escherichia coli
**stec**	pathogen	Shiga toxin-producing Escherichia coli

### 4.4 Enteric pathogens and virulence factor prevalence

The abundance of 23 enteric pathogen markers was assessed using qPCR. All children had between 1 to 15 pathogen or virulence factors detected in at least one visit (figure 3b). In accordance with microbial richness, we observed MAM to have a greater number of pathogenic markers (8 pathogens) compared to SAM or WN (7 pathogens) nutritional status phenotypes. Several pathogenic markers were differentially prevalent between the nutritional status phenotypes. In particular, *Giardia duodenalis* was significantly less prevalent among children with SAM (23%) than MAM (68%) or WN (70%) (Fisher's exact test; adjusted p = < 0.001). In contrast, the prevalence of enteropathogenic *E. coli* with the *bfpA* (adjusted p = 0.007) and *eae* (adjusted p = 0.024) virulence increased with severity of malnutrition (figure 3b). Bundle-forming pilus A [*bfpA*] and intimin adherence protein [*eae*] are genes found on the EAF plasmid and EPEC genome, respectively, and contribute to attachment to epithelial cells, thus, leading to the attaching and effacing phenotype [60,61].

### 4.5 Identifying perturbations capable of discriminating nutritional status phenotypes

Our SSPN analysis produced a perturbation matrix (N= 82 samples, M= 14,270 edges representing 188 nodes) (supplementary figure 5). By analyzing our SSPNs using a HEC model and feature selection in unison, the most accurately predictive edges within the network were identified. Essentially, this characterizes which interactions are most predictive of nutritional phenotype and is more informative for nutritional intervention than regression methods predicting a rise or fall in WHZ and disregarding intra-phenotype patterns. By selecting for only the edges that are informative in discriminating nutritional status phenotypes, and by extension the informative nodes, the information content in the edges was compressed by 98.143%; that is, 265 of the 14,270 edges.

**Figure 4:**
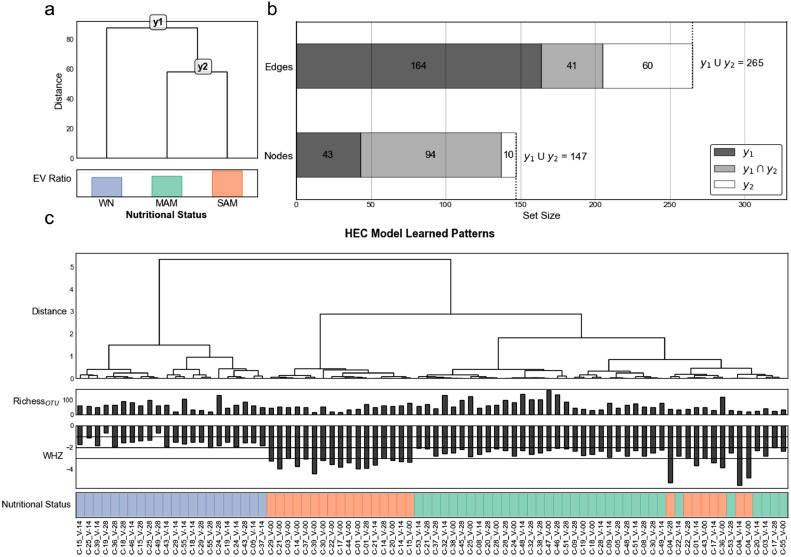
Hierarchical classification scheme for phenotypes using sample-specific perturbation networks, (a) Data-driven HEC model structure determined from perturbation matrix. (b) Barchart-styled Venn diagram showing node and edge sets selected by Clairvoyance. (c) Unsupervised clustering of prediction probabilities from HEC model on held-out LSOCV test sets.

Unsupervised machine-learning can be used to gain insight into the underlying structure of the data and leveraged to validate dimensionality reduction (e.g. feature selection) results based on predefined categories, nutritional status in this context. Unsupervised clustering of held-out prediction probabilities (figure 4c) were more homogenous than unsupervised clustering based on microbiome abundance profiles (figure 2c) or pathogen markers (supplementary figure 3). Clustering by held-out prediction probabilities revealed that the edge features in each sub-model capture biologically relevant discriminatory patterns. It should be noted that the unsupervised clustering in figure 4c used Euclidean distance of probabilities generated from multimodal edges while figure 2c and supplementary figure 3 used Euclidean distance of individual modalities. We were not able to implement unsupervised clustering of the multimodal nodes using Euclidean distances because the pathogenic markers are binary and clinical measurements had missing data.

### 4.6 Connectivity signatures in aggregate networks

**Figure 5:**
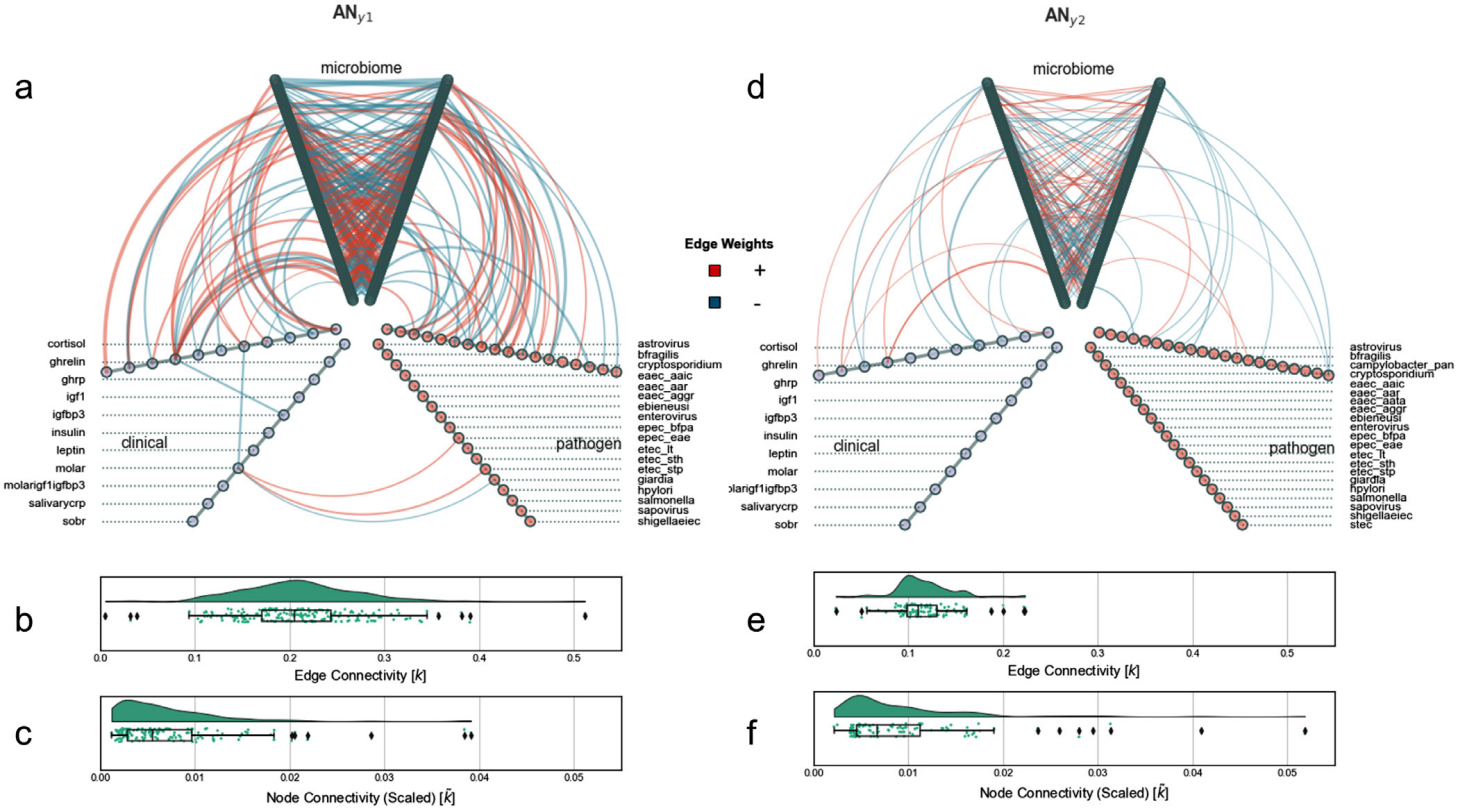
Hive plot network visualization of aggregate networks, Aggregate networks built using edges selected by Clairvoyance with edge weights from fitted logistic regression for sub-models y1 and y2 corresponding to AN_y1_ and AN_y2,_ respectively. Hive plots of (a) AN_y1_ and (d) AN_y2_ to visualize intra-modality and inter-modality connections where each axis represents a particular modality. Raincloud plots showing edge connectivity (b, e) and scaled node connectivity (c,f) of AN_y1_ and AN_y2_, respectively.

We implemented aggregate networks [AN] from each sub-model to structure the edge perturbations that could accurately predict nutritional status phenotype (figure 5). Edge weight in the context of ANs corresponds with the capacity of a specific edge to predict nutritional status phenotype and these networks serve as a method to quantify the predictive capacity of specific nodes and edges for clinical interpretation. As the training data for the parent sub-models are edge perturbations with respect to a sample in a SSPN, an increase in association strength (a positive perturbation) and a positive coefficient indicate an increased likelihood for said classes (See *Methods*).

The higher scaled node connectivity (Mann-Whitney rank test, p = < 0.001) and lower edge connectivity (Mann-Whitney rank test, p < 0.001) in ***AN_y2_*** revealed there were a few nodes with high predictive capacity compared to ***AN_y1_***which was closer to a normal distribution. In ***AN_y1_***, we observed an unclassified *Escherichia-Shigella* (*Otu000281*)*,* as the one of two nodes with substantially more influence in discriminating WN from malnourished children. The highest weighted edges to this unclassified *Escherichia-Shigella* were through *ghrelin* (-0.321 *k*) and *ghrp* (0.311 *k*) (supplementary figure 6a). The second node with substantially higher influence in ***AN_y1_*** was *molar* (0.0384 *k^∼^*). The highest connectivity edges to *molar* were through *Lactobacillus mucosae*, an unclassified *Haemophilus* (0.289 *k*), and an unclassified *Ruminococcaceae* UCG-002 (0.264 *k*) (supplementary figure 6b). The only edge in *AN_y1_* with substantially greater edge connectivity relative to the other edges in the network was *Otu000965*:unclassified *Pantoea* — *Otu000906:Bifidobacterium stellenboschense* (-0.483 *k*).

The scaled node connectivity in *AN_y2_* was greater than *AN_y1_* and this difference was largely influenced by two outlier *Enterobacteriaceae* nodes with much higher connectivity relative to the other nodes in the network: (1) an unclassified *Pantoea* (0.0519 *k^∼^*) and (2) an unclassified *Enterobacteriaceae* (0.0409 *k^∼^*). Therefore, these two nodes accumulated 9.27% of the total connectivity within *AN_y2_*. When grouping nodes by family-level taxonomy, we observed that *Enterobacteriaceae* constituted 14.61% of the total connectivity in *AN_y2_*. The greater edge connectivity in *AN_y2_* was largely influenced by four high connectivity edges: (1) *Weissella cibaria* — *Lactobacillus oris* F0423 (-0.222 *k*)*;* (2) *igf1* — unclassified *Enterobacteriaceae* (-0.220 *k*); (3) unclassified *Streptococcus* —unclassified *Megasphaera*, (-0.201 *k***)**; and (4) *Prevotella* — *Subdoligranulum* (0.191 *k*).

### 4.7 Quantifying changes in sample-specific perturbation networks over time

**Figure 6:**
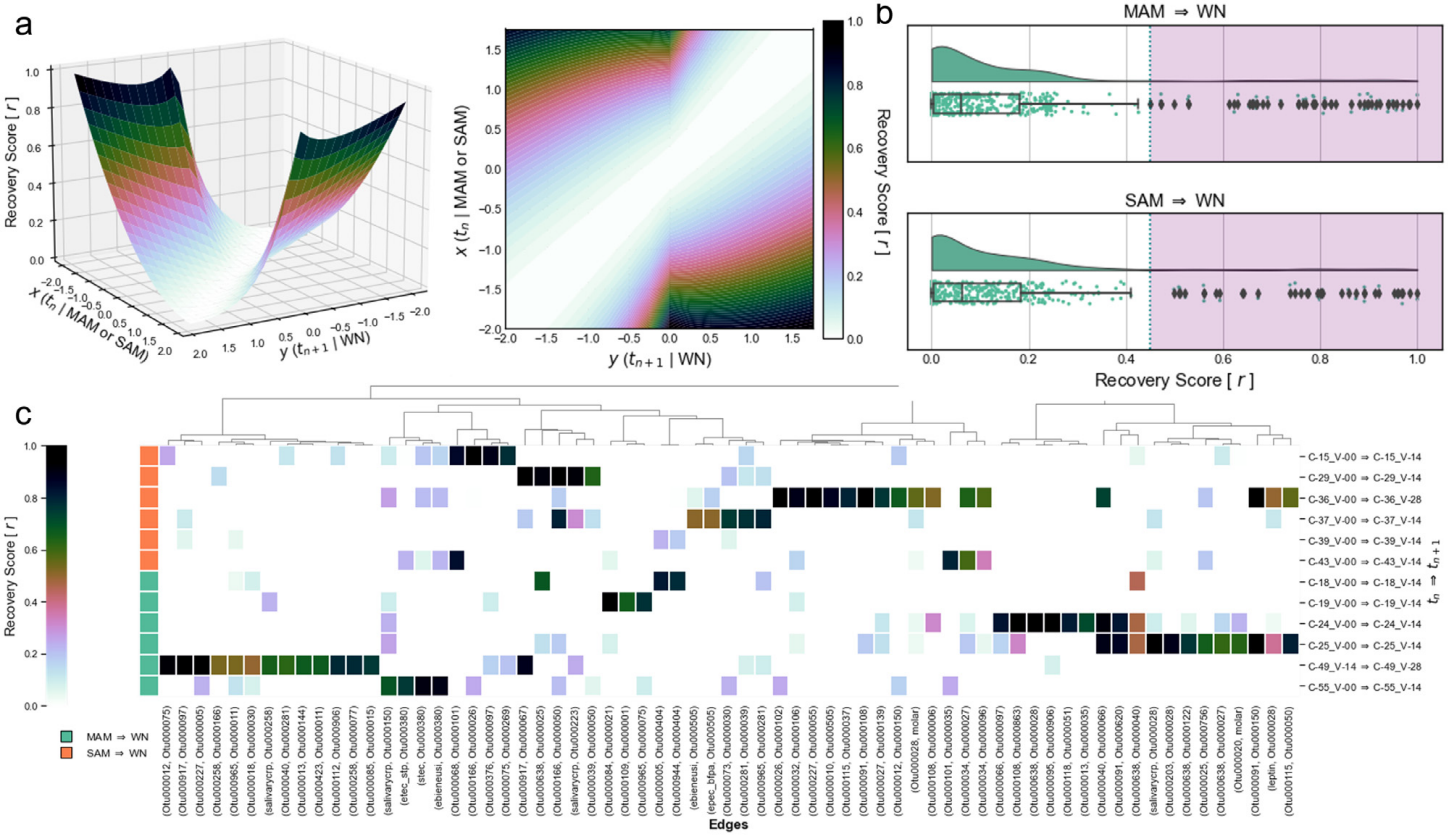
Recovery score for time-ordered SSPNs, (a) Domain and topology of recovery score in (left) 3-dimensions and (right) 2-dimensions. (b) Distributions of recovery scores for (top) MAM → WN and (bottom) SAM → WN transitions with boxplot whiskers representing IQR and violet overlay showcasing outlier regions (IQR*1.5 + Q3). (c) Clustermap showcasing recovery scores for outlier edges indicated in (b) and organized by transitional event.

Time-ordered SSPNs provide unique insight into how these multimodal systems evolve over time in relation to nutritional status recovery. We developed the recovery score [***r***] to reduce the information content of time-ordered SSPNs and rank edges by their potential contribution to nutritional status recovery. The recovery score is designed to reward edges with large differences between ***t_n_*** and ***t_n+1_*** while penalizing larger values of ***t_n+1_***where ***t_n_*** and ***t_n+1_***represent edge weights for UN and WN consecutive visits, respectively. This property of the recovery score can be visualized with the convex shape defined by ***r(x,y)*** as lower scores exist when ***t_n_*** and ***t_n+1_***are similar or when ***t_n+1_*** is large (figure 6a).

There were a total of 67 edges representing 76 unique nodes from 12 participants that had recovery scores statistically greater than the IQR (*r_threshold_* ≥ 0.449). The 12 participants in this statistically significant group are represented by 6 MAM to WN transitions (MAM → WN) and 6 SAM to WN transitions (SAM → WN). The 76 nodes from edges with statistically significant recovery scores included 69 OTUs (40 *Firmicutes*, 12 *Proteobacteria*, 12 *Bacteriodetes*, 4 *Actinobacteria*, and 1 *Fusobacteria*), 3 clinical measurements (*leptin, molar, salivarycrp*), and 4 enteric pathogens (*ebieneusi, epec_bfpa, etec_stp, stec*). Unsupervised clustering of statistically significant recovery scores did not reveal any noticeable groupings by MAM → WN or SAM → WN transitions (figure 6c). However, one group of transitions (N=2 MAM → WN and N=1 SAM → WN) with participants aged from 20.3 – 24.4 months formed a tight cluster outside of the remaining 9 transitions. Both MAM samples in these transitions have WHZ ≤ -2.79, close to the -3 WHZ threshold for SAM, which suggests a similar nutritional status to the lone SAM → WN transition in the cluster.

Many outlier recovery scores were unique to a participant within our cohort. However, there were a few exceptions where we observed more consistency across participants with similar nutritional status shifts. In particular, 3 edges had statistically high recovery scores in multiple MAM → WN transitions including OTUs belonging to *Prevotellaceae, Lachnospiraceae, Phascolarctobacterium and Ruminococcaceae* species. We also observed 3 edges that had statistically high recovery scores in more than one SAM → WN transition including *Lachnospiraceae, Coriobacteriaceae, Prevotellaceae, Subdoligranulum and Bacteroides* and *Enterobacteriaceae* species*.* The cohort sample size was not large enough to statistically model specific associations in a participant's ability to decline or recover with respect to their nutritional status.

## 5 Discussion

In this study, we have shown that children with non-edematous SAM (marasmus) that is the more prevalent type in this region of The Gambia, have distinct microbiome characteristics and biologically-relevant multimodal biomarkers not observed in MAM and WN nutritional phenotypes. These findings also provide some key preliminary insights into systematic changes in gut microbiota and host energy regulating hormones among children with marasmus SAM during outpatient nutritional rehabilitation and among under 5’s with marasmus SAM, MAM and WN nutritional phenotypes, that may have important implications for future research into prevention and treatment strategies of acute malnutrition [24].

Analyzing each data type (modality) independently allowed us to validate our findings by cross-referencing against previous studies and set the context for our multimodal network analysis. Consistent with previous reports [12,13,15,62], we found that children with SAM had significant reductions in richness and bacterial loads compared to WN or MAM participants. It is possible that the bacteria depleted in acutely malnourished children are essential for optimal digestion, nutrient absorption, modulating inflammation and immune development [12]. A new finding from our study was that children with MAM had statistically significant enrichments in the gut bacterial loads and variance in microbial richness compared to WN and SAM participants. These changes may be an indication of impaired immune function in the children with acute malnutrition [63] which agrees with our findings that IGF-1 and leptin are associated with various microbes and are highly predictive of nutritional status.

The aggregate network analyses suggest that WN and SAM are relatively more stable ecosystems and as a child transitions from WN to SAM they encounter a more chaotic and unpredictable microbiome. In addition to SAM having lower microbial diversity than MAM and WN, we also observed an enrichment in *Enterobacteriaceae* abundance therefore increasing the risk of adverse outcomes. Previous metagenomics studies have reported reduced microbial diversity and an increase in pathogenic *Enterobacteriaceae* among malnourished children [64].

Analysis of clinical measurements pertaining to energy regulating hormones gave insight into which metabolites were proportional to fluctuations in WHZ. We identified statistically significant trends between IGF-1, leptin, and IGFBP- 3 (the main binding protein of IGF-1) that increase with WHZ and nutritional status. IGF-1 is a key growth regulating hormone in infancy and plays an important role during nutritional recovery in undernourished children. Similarly, leptin is a hormone predominantly made by adipose cells and enterocytes in the small intestine to help regulate energy balance by moderating appetite and intestinal barrier function [65] and plays a major role in signaling energy deficit in acute malnutrition (Bouillanne et al., 2007; Prentice, Moore, Collinson, & O'Connell, 2002). We also observed reverse trends in that the levels of sOB-R (and the molar excess of sOB-R:leptin), ghrelin (and its binding protein) increase monotonically as WHZ decreases. Our findings support the hypothesis by Stein et al. 2006 that sOB-R is upregulated during starvation to maintain low levels of bioactive leptin and increase its half-life, thus, decreasing energy expenditure and increasing food uptake. Ghrelin is a well-studied hormone produced by enteroendocrine cells of the gastrointestinal, tract with substantial production in the stomach, [66,67] and circulating ghrelin blood levels are often highest when an individual experiences hunger while returning to lower levels after food intake [67,68]. Our clinical measurements of these metabolites are consistent with previous studies and provide a strong foundation for more complex analytical methods in the context of acute malnutrition.

*Enterobacteriaceae* abundance is greatly enriched in SAM children and may be linked to the low prevalence of *Giardia* which competes for the same ecological niche in the small intestine [69]. However, previous research using mouse models showed that Enterobacteriaceae is over-representation in Giardia infected mice [70]. There appears to be a more complex mechanism regulating the balance between Enterobacteriaceae (bacteria) and Giardia (protozoan parasite). Our finding may therefore be specific for marasmic SAM involving the differential regulation of anti-parasitic and anti-bacterial immune responses in these children where Giardia infection alters immune responses to *E.coli* or vice versa. This warrants further exploration. We also observed that EPEC virulence factors *bfpA* and *eae* increased with increasing severity of malnutrition. EPEC adheres to intestinal epithelial cells, causing diarrhea, and constitutes a significant risk to health, especially in very young children [71]. Subramanian and colleagues also reported an enrichment of *Enterobacteriaceae spp.* among children with SAM from Bangladesh [9,13]; although, a causal pathway is yet to be identified.

We found that *Escherichia/Shigella* sp. and molar ratio of sOB-R:letpin had substantially greater predictive capacity in discriminating WN from undernourished participant samples compared to other nodes. In particular, this *Escherichia-Shigella* OTU had high predictive capacity through its associations with *ghrelin* and *ghrp*. This is not surprising as leptin is a key player in regulating both antimicrobial peptides and microbiota composition and as such, *Escherichia-Shigella* and molar-excess soluble leptin may play pivotal roles in mediating complex interactions that modulate nutritional status. In the TAC analyses, the gene target for *Escherichia/Shigella* was *ipaH* which is carried by *Shigella* sp. and EIEC. However, it should be noted that the signals from the *ipaH* gene target detected are most likely to have come from *Shigella* and not EIEC. Our previous studies showed that *Shigella flexneri* and *Shigella sonnei* account for the majority of ipaH detections[36].

We observed high predictive capacity of molar excess of sOB-R:leptin through *Lactobacillus mucosae,* an unclassified *Haemophilus* and an unclassified *Ruminococcaceae* UCG-002. Previous research has identified strong associations between leptin and *Lactobacillus* and it is believed that leptin can modulate gut microbiota by stimulating mucin production which may favor bacterial growth [72]. *Lactobacillus* has been shown to maintain intestinal homeostasis and is speculated to attenuate the pro-inflammatory signaling induced by *Shigella* after invasion of epithelial lining [73]. Similarly, previous research has ascertained that leptin supplementation resulted in a higher proportion of *Ruminococcaceae* [74]. To our knowledge, no research has investigated relationships between *Haemophilus* and leptin in the context of acute malnutrition. Another intriguing finding was the high predictive capacity of perturbations in *IGF-1* and an unclassified *Enterobacteriacea* in discriminating MAM from SAM*.* The high predictive capacity of perturbations in *IGF-1* and the *Enterobacteriaceae* associations are relevant as *Enterobacteriaceae* are often enriched in children who are wasted along with decreased plasma IGF-1 concentrations [75] and decreased concentrations of IGF-1 and IGFBP- 3 have been observed in underweight mice [76]. *Enterobacteriaceae* are often be enriched in undernourished individuals [64] and coupled with decreased concentrations of IGF-1 [75] and IGFBP- 3 [76]. These findings from other research groups agree with our results showing that IGF-1 and IGFBP- 3 concentrations decrease with WHZ and are lowest in SAM. As immunity is heavily impaired in children experiencing SAM [77], the predictive associations between *Enterobacteriacea* and IGF-1 are not surprising. However, it is not uncommon for children experiencing SAM to develop septicaemia [63,77]. Previous research has shown that patients with sepsis have low levels of IGF-1 inversely correlated with enteric bacterial load [78]. Hunninghake et al. 2010 also supposed that translocation of bacteria across the gastrointestinal tract may occur.

Our predictive functional profiling analysis found that phenylalanine metabolism and benzoate degradation pathways were enriched in SAM compared to WN. Several prior studies have investigated phenylalanine in the context of undernutrition in early childhood [79]. To our knowledge, this is the first indication that benzoate degradation may play a significant role in acute malnutrition but this needs further investigation. Other individual enzymes that appear to have been lost to some degree in the SAM condition that could influence host nutrition include sortase, tryptophanase, and butyrate kinase. While far more single enzymes are enriched in SAM, they are only sparingly enriched relative to those that have been lost. Finally, these functional results are merely predictions based on a computational tool [45], thus should be considered carefully without further validation using shotgun metagenomic analyses.

Acute malnutrition is a complex multifactorial disease with interplay between the gut microbiome, energy regulating hormones, and the presence of enteric pathogens. It appears that WN systems are stable but as a child's weight declines, approaching MAM, the community destabilizes with increased microbial diversity and interactions. As a child's nutritional status deteriorates the gut microbiota community becomes depleted and dominated by pathogenic *Enterobacteriaceae* in an ecological collapse as demonstrated by low bacterial load, and low microbial diversity. Using novel methods, we show there are potentially diagnostic interactions for each of these transitions. The methods introduced in this study build upon the existing SSN framework of Liu et al. 2016 to investigate patient-specific networks by extending an approach into a multimodal framework and by bootstrapping samples to obtain distributions of associations rather than single point values. This work not only provides an insight into dynamic multimodal systems in the context of acute malnutrition but also illuminates the potential avenues for diagnostics and therapeutics. The framework and methods introduced in this research can applied broadly across biological sciences.

## Contributors

HMN, BAK, MA and AMP conceptualized and designed the study; HMN, MC coordinated the data collection; ATJ, RSB processed the stool samples; JL and ERH provided TAC array cards and interpretation, BAK, MA, JS, MB, RB, CO and AKS coordinated and undertook the enteric pathogen analyses and microbiome sequencing; JLE and CLD performed microbiome analysis, network analysis, software development, and statistical modelling. JLE, HMN, CLD and BAK verified the underlying data, drafted the initial manuscript; all authors reviewed, revised, and approved the final version of the manuscript.

We would like to thank the MRCG@LSHTM Nutrition Theme field and laboratory teams that supported the collection and processing of the specimens. Our thanks are also due to all the study participants and their families. We would also like to acknowledge Naisha Shah of *J. Craig Venter Institute* for her insight into multimodal sample-specific network analysis.

## Data Sharing Statement

Materials (clinical and biological specimens) will be shared upon request for health research provided there are sufficient quantities, appropriate agreements and ethical approvals in place. Further information and requests for resources and reagents should be directed to and will be fulfilled by the lead contacts Brenda A. Kwambana-Adams (brenda.kwambana@ucl.ac.uk) and Christopher L. Dupont (cdupont@jcvi.org).

The 16S amplicon reads generated during this study are available at NCBI via SRA:SRR 14459253- 14459364 (BioSamples: SAMN19053066-19053178) under BioProject PRJNA727842. The code, methodologies, and tutorials for Ensemble Networks are open-sourced in our *EnsembleNetworkX* Python package (https://github.com/jolespin/ensemble_networkx) under the BSD-3 license. Metadata, datasets, and networks are available via https://doi.org/10.6084/m9.figshare.16733584.

## Declaration of Competing Interest

The authors have no competing interests to declare.

## Appendix Supplementary materials

Image, application 1

Image, application 2

## References

[1] Forbes GB. (1974). Joint FAO/WHO ad hoc Expert Committee, Energy and Protein Requirements, WHO Technical Report Series 522. Arch Pediatr Adolesc Med.

[2] Black RE, Victora CG, Walker SP, Bhutta ZA, Christian P, De Onis M (2013). Maternal and child undernutrition and overweight in low-income and middle-income countries. Lancet.

[3] World Health Organization (2006). WHO child growth standards: length/height-for-age, weight-for-age, weight-for-length. weight-for-height and body mass index-for-age: methods and development.

[4] Franco VHM, Hotta JKS, Jorge SM, Dos Santos JE (1999). Plasma fatty acids in children with grade III protein-energy malnutrition in its different clinical forms: Marasmus, marasmic kwashiorkor, and kwashiorkor. J Trop Pediatr.

[5] The Gambia Bureau of Statistics (2019). The Gambia Multiple Indicator Cluster Survey 2018. Banjul.

[6] (2018). National Nutrition Agency (NaNA)-Gambia, UNICEF GB of S. Gambia National Micronutrient Survey. Banjul.

[7] Velly H, Britton RA, Preidis GA. (2017). Mechanisms of cross-talk between the diet, the intestinal microbiome, and the undernourished host. Gut Microbes.

[8] Nabwera HM, Fulford AJ, Moore SE, Prentice AM. (2017). Growth faltering in rural Gambian children after four decades of interventions: a retrospective cohort study. Lancet Glob Heal.

[9] Platts-Mills JA, Taniuchi M, Uddin MJ, Sobuz SU, Mahfuz M, Gaffar SMA (2017). Association between enteropathogens and malnutrition in children aged 6-23 mo in Bangladesh: A case-control study. Am J Clin Nutr.

[10] Richard SA, McCormick BJJ, Miller MA, Caulfield LE, Checkley W (2014). MAL-ED Network Investigators. Modeling environmental influences on child growth in the MAL-ED cohort study: opportunities and challenges. Clin Infect Dis.

[11] Keusch GT, Denno DM, Black RE, Duggan C, Guerrant RL, Lavery JV (2014). Environmental enteric dysfunction: Pathogenesis, diagnosis, and clinical consequences. Clin Infect Dis.

[12] Smith MI, Yatsunenko T, Manary MJ, Trehan I, Mkakosya R, Cheng J (2013). Gut microbiomes of Malawian twin pairs discordant for kwashiorkor. Science.

[13] Subramanian S, Huq S, Yatsunenko T, Haque R, Mahfuz M, Alam MA (2014). Persistent gut microbiota immaturity in malnourished Bangladeshi children. Nature.

[14] Kristensen KHS, Wiese M, Rytter MJH, Özçam M, Hansen LH, Namusoke H (2016). Gut Microbiota in Children Hospitalized with Oedematous and Non-Oedematous Severe Acute Malnutrition in Uganda. PLoS Negl Trop Dis.

[15] Gough EK, Stephens DA, Moodie EEM, Prendergast AJ, Stoltzfus RJ, Humphrey JH (2015). Linear growth faltering in infants is associated with Acidaminococcus sp. and community-level changes in the gut microbiota. Microbiome.

[16] Yatsunenko T, Rey FE, Manary MJ, Trehan I, Dominguez-Bello MG, Contreras M (2012). Human gut microbiome viewed across age and geography. Nature.

[17] Yadav D, Ghosh TS, Mande SS. (2016). Global investigation of composition and interaction networks in gut microbiomes of individuals belonging to diverse geographies and age-groups. Gut Pathog.

[18] Davis JCC, Lewis ZT, Krishnan S, Bernstein RM, Moore SE, Prentice AM (2017). Growth and Morbidity of Gambian Infants are Influenced by Maternal Milk Oligosaccharides and Infant Gut Microbiota. Sci Rep.

[19] Kashyap PC, Chia N, Nelson H, Segal E, Elinav E. (2017). Microbiome at the Frontier of Personalized Medicine. Mayo Clin. Proc..

[20] David LA, Maurice CF, Carmody RN, Gootenberg DB, Button JE, Wolfe BE (2014). Diet rapidly and reproducibly alters the human gut microbiome. Nature.

[21] Grzeskowiak L, Collado MC, Mangani C, Maleta K, Laitinen K, Ashorn P (2012). Distinct Gut microbiota in southeastern African and northern European infants. J Pediatr Gastroenterol Nutr.

[22] De Filippo C, Cavalieri D, Di Paola M, Ramazzotti M, Poullet JB, Massart S (2010). Impact of diet in shaping gut microbiota revealed by a comparative study in children from Europe and rural Africa. Proc Natl Acad Sci U S A.

[23] Monira S, Nakamura S, Gotoh K, Izutsu K, Watanabe H, Alam NH (2011). Gut microbiota of healthy and malnourished children in Bangladesh. Front Microbiol.

[24] Blanton LV., Barratt MJ, Charbonneau MR, Ahmed T, Gordon JI (2016). Childhood undernutrition, the gut microbiota, and microbiota-directed therapeutics. Science.

[25] Chen RY, Mostafa I, Hibberd MC, Das S, Mahfuz M, Naila NN (2021). A Microbiota-Directed Food Intervention for Undernourished Children. N Engl J Med.

[26] Mostafa I, Nahar NN, Islam MM, Huq S, Mustafa M, Barratt M (2020). Proof-of-concept study of the efficacy of a microbiota-directed complementary food formulation (MDCF) for treating moderate acute malnutrition. BMC Public Health.

[27] Gehrig JL, Venkatesh S, Chang HW, Hibberd MC, Kung VL, Cheng J (2019). Effects of microbiota-directed foods in gnotobiotic animals and undernourished children. Science.

[28] Lunn PG, Erinoso HO, Northrop-Clewes CA, Boyce SA. (1999). Giardia intestinalis is unlikely to be a major cause of the poor growth of rural Gambian infants. J Nutr.

[29] Campbell DI, McPhail G, Lunn PG, Elia M, Jeffries DJ. (2004). Intestinal inflammation measured by fecal neopterin in Gambian children with enteropathy: association with growth failure, Giardia lamblia, and intestinal permeability. J Pediatr Gastroenterol Nutr.

[30] Thomas JE, Gibson GR, Darboe MK, Weaver LT, Dale A. (1992). Isolation of Helicobacter pylori from human faeces. Lancet.

[31] (2020). World Food Programme. WFP The Gambia - Country Brief.

[32] Husseini M, Darboe MK, Moore SE, Nabwera HM, Prentice AM. (2018). Thresholds of socio-economic and environmental conditions necessary to escape from childhood malnutrition: A natural experiment in rural Gambia. BMC Med.

[33] Nabwera HM, Bernstein RM, Agbla SC, Moore SE, Darboe MK, Colley M (2018). Hormonal Correlates and Predictors of Nutritional Recovery in Malnourished African Children. J Trop Pediatr.

[34] World Health Organization (2013). Pocket Book of Hospital Care for Children: Guidelines for the Management of Common Childhood Illnesses. World Health Organization.

[35] Stein K, Vasquez-Garibay E, Kratzsch J, Romero-Velarde E, Jahreis G. (2006). Influence of Nutritional Recovery on the Leptin Axis in Severely Malnourished Children. J Clin Endocrinol Metab.

[36] Liu J, Gratz J, Amour C, Kibiki G, Becker S, Janaki L (2013). A laboratory-developed taqman array card for simultaneous detection of 19 enteropathogens. J Clin Microbiol.

[37] Donnenberg MS, Girón JA, Nataro JP, Kaper JB. (1992). A plasmid-encoded type IV fimbrial gene of enteropathogenic Escherichia coli associated with localized adherence. Mol Microbiol.

[38] Edgar RC. (2013). UPARSE: Highly accurate OTU sequences from microbial amplicon reads. Nat Methods.

[39] Schloss PD, Westcott SL, Ryabin T, Hall JR, Hartmann M, Hollister EB (2009). Introducing mothur: Open-source, platform-independent, community-supported software for describing and comparing microbial communities. Appl Environ Microbiol.

[40] Price MN, Dehal PS, Arkin AP. (2010). FastTree 2–approximately maximum-likelihood trees for large alignments. PLoS One.

[41] Morton JT, Sanders J, Quinn RA, McDonald D, Gonzalez A, Vázquez-Baeza Y (2017). Balance Trees Reveal Microbial Niche Differentiation. mSystems.

[42] Biocore (2020). scikit-bio: A Bioinformatics Library for Data Scientists, Students, and Developers. GitHub.

[43] Espinoza JL. (2020). compositional: Compositional data analysis in Python. GitHub.

[44] Virtanen P, Gommers R, Oliphant TE, Haberland M, Reddy T, Cournapeau D (2020). SciPy 1.0: fundamental algorithms for scientific computing in Python. Nat Methods.

[45] Douglas GM, Maffei VJ, Zaneveld JR, Yurgel SN, Brown JR, Taylor CM (2020). PICRUSt2 for prediction of metagenome functions. Nat. Biotechnol..

[46] Sun S, Jones RB, Fodor AA. (2020). Inference-based accuracy of metagenome prediction tools varies across sample types and functional categories. Microbiome.

[47] Subramanian A, Tamayo P, Mootha VK, Mukherjee S, Ebert BL, Gillette MA (2005). Gene set enrichment analysis: A knowledge-based approach for interpreting genome-wide expression profiles. Proc Natl Acad Sci U S A.

[48] Fernandes AD, Macklaim JM, Linn TG, Reid G, Gloor GB. (2013). ANOVA-Like Differential Expression (ALDEx) Analysis for Mixed Population RNA-Seq. PLoS One.

[49] Espinoza JL. (2019). soothsayer: High-level analysis package for (bio-)informatics. GitHub.

[50] Seabold S, Perktold J. (2010). Statsmodels: Econometric and Statistical Modeling with Python.

[51] Espinoza JL, Shah N, Singh S, Nelson KE, Dupont CL. (2020). Applications of weighted association networks applied to compositional data in biology. Environ Microbiol.

[52] Erb I, Notredame C. (2016). How should we measure proportionality on relative gene expression data?. Theory Biosci.

[53] Lovell D, Pawlowsky-Glahn V, Egozcue JJ, Marguerat S, Bähler J. (2015). Proportionality: A Valid Alternative to Correlation for Relative Data. PLOS Comput Biol.

[54] Hagberg AA, Schult DA, Swart PJ. (2008). Exploring Network Structure, Dynamics, and Function using NetworkX.

[55] Espinoza JL. (2020). hive_networkx: Hive plots in Python. GitHub.

[56] Krzywinski M, Birol I, Jones SJ, Marra MA. (2012). Hive plots–rational approach to visualizing networks. Brief Bioinform.

[57] Allen M, Poggiali D, Whitaker K, Marshall TR, Kievit RA. (2019). Raincloud plots: A multi-platform tool for robust data visualization. Wellcome Open Res.

[58] Espinoza JL, Dupont CL, O'Rourke A, Beyhan S, Morales P, Spoering A (2021). Predicting antimicrobial mechanism-of-action from transcriptomes: A generalizable explainable artificial intelligence approach. PLOS Comput Biol.

[59] Dreiseitl S, Ohno-Machado L. (2002). Logistic regression and artificial neural network classification models: A methodology review. J Biomed Inform.

[60] Blank TE, Zhong H, Bell AL, Whittam TS, Donnenberg MS. (2000). Molecular variation among type IV pilin (bfpA) genes from diverse enteropathogenic Escherichia coli strains. Infect Immun.

[61] Slinger R, Lau K, Slinger M, Moldovan I, Chan F. (2017). Higher atypical enteropathogenic Escherichia coli (a-EPEC) bacterial loads in children with diarrhea are associated with PCR detection of the EHEC factor for adherence 1/lymphocyte inhibitory factor A (efa1/lifa) gene. Ann Clin Microbiol Antimicrob.

[62] Alou MT, Million M, Traore SI, Mouelhi D, Khelaifia S, Bachar D (2017). Gut bacteria missing in severe acute malnutrition, can we identify potential probiotics by culturomics?. Front Microbiol.

[63] Jones KDJ, Berkley JA. (2014). Severe acute malnutrition and infection. Paediatr Int Child Health.

[64] Million M, Diallo A, Raoult D. (2017). Gut microbiota and malnutrition. Microb. Pathog..

[65] Brennan AM, Mantzoros CS. (2006). Drug Insight: The role of leptin in human physiology and pathophysiology - Emerging clinical applications. Nat. Clin. Pract. Endocrinol. Metab..

[66] Kojima M, Hosoda H, Date Y, Nakazato M, Matsuo H, Kangawa K. (1999). Ghrelin is a growth-hormone-releasing acylated peptide from stomach. Nature.

[67] Müller TD, Nogueiras R, Andermann ML, Andrews ZB, Anker SD, Argente J (2015). Ghrelin. Mol. Metab..

[68] Cummings DE, Purnell JQ, Frayo RS, Schmidova K, Wisse BE, Weigle DS. (2001). A Preprandial Rise in Plasma Ghrelin Levels Suggests a Role in Meal Initiation in Humans. Diabetes.

[69] Allain T, Amat CB, Motta JP, Manko A, Buret AG. (2017). Interactions of Giardia sp. with the intestinal barrier: Epithelium, mucus, and microbiota. Tissue Barriers.

[70] Bartelt LA, Bolick DT, Mayneris-Perxachs J, Kolling GL, Medlock GL, Zaenker EI (2017). Cross-modulation of pathogen-specific pathways enhances malnutrition during enteric co-infection with Giardia lamblia and enteroaggregative Escherichia coli. PLOS Pathog.

[71] Deborah Chen H, Frankel G (2005). Enteropathogenic Escherichia coli: Unravelling pathogenesis. FEMS Microbiol. Rev..

[72] El Homsi M, Ducroc R, Claustre J, Jourdan G, Gertler A, Estienne M (2007). Leptin modulates the expression of secreted and membrane-associated mucins in colonic epithelial cells by targeting PKC, PI3K, and MAPK pathways. Am J Physiol - Gastrointest Liver Physiol.

[73] Tien M-T, Girardin SE, Regnault B, Le Bourhis L, Dillies M-A, Coppée J-Y (2006). Anti-Inflammatory Effect of Lactobacillus casei on Shigella -Infected Human Intestinal Epithelial Cells. J Immunol.

[74] Grases-Pintó B, Abril-Gil M, Castell M, Rodríguez-Lagunas MJ, Burleigh S, Fåk Hållenius F (2019). Influence of Leptin and Adiponectin Supplementation on Intraepithelial Lymphocyte and Microbiota Composition in Suckling Rats. Front Immunol.

[75] Bartz S, Mody A, Hornik C, Bain J, Muehlbauer M, Kiyimba T (2014). Severe acute malnutrition in childhood: Hormonal and metabolic status at presentation, response to treatment, and predictors of mortality. J Clin Endocrinol Metab.

[76] Schwarzer M, Makki K, Storelli G, Machuca-Gayet I, Srutkova D, Hermanova P (2016). Lactobacillus plantarum strain maintains growth of infant mice during chronic undernutrition. Science.

[77] Hossain MI, Chisti J, Yoshimatsu S, Yasmin R, Ahmed T (2015). Features in Septic Children With or Without Severe Acute Malnutrition and the Risk Factors of Mortality. Pediatrics.

[78] Hunninghake GW, Doerschug KC, Nymon AB, Schmidt GA, Meyerholz DK, Ashare A. (2010). Insulin-like growth factor-1 levels contribute to the development of bacterial translocation in sepsis. Am J Respir Crit Care Med.

[79] Jahoor F, Badaloo A, Reid M, Forrester T. (2006). Sulfur amino acid metabolism in children with severe childhood undernutrition: Methionine kinetics. Am J Clin Nutr.

[80] Liu X, Wang Y, Ji H, Aihara K, Chen L. (2016). Personalized characterization of diseases using sample-specific networks. Nucleic Acids Res.

[81] Nataro JP, Martinez J. (1998). Diagnosis and Investigation of Diarrheagenic Escherichia coli. Methods Mol Med.

[82] J H, AG T (2015). Enteropathogenic Escherichia coli: foe or innocent bystander?. Clin Microbiol Infect.

[83] Asea A, Kaur P, Chakraborti A. (2010). Enteroaggregative Escherichia coli: An emerging enteric food borne pathogen. Interdiscip. Perspect. Infect. Dis..

[84] Fleckenstein JM, Kuhlmann FM. (2019). Enterotoxigenic Escherichia coli Infections. Curr. Infect. Dis. Rep..

