# Supplementary material for: Interactions between fecal gut microbiome, enteric pathogens, and energy regulating hormones among acutely malnourished rural Gambian children
Source: eBioMedicine. 2021 Oct 22;73:103644. doi: 10.1016/j.ebiom.2021.103644 (PMC8550991; doi:10.1016/j.ebiom.2021.103644)
Supplement: Supplementary file 1 [file mmc1.docx]

**Supplemental Information:**

*Figure S1 – Number of detected OTUs in relation to the sequencing depth*

Shows the number of total reads mapped to OTUs in log-scale vs. the total number of detected OTUs per sample.

*Figure S2 – Microbial richness, variance, and WHZ relationships*

Shows various measurements at the (a) family, (b) genus, (c) species, and (d) OTU levels.

(e) Shows the WHZ trajectory of participants with red and blue indicating a decrease and increase in WHZ, respectively, between subsequent visits.

*Figure S3 – Clustermap of enteric pathogen markers*

Hierarchical clustering with heatmap showing presence and absence of enteric pathogens for each sample.

*Figure S4 – Linear mixed-effects models significant covariates*

Directed network showing significant covariates associated with fixed effects. Weighted edges connecting nodes are positive and negative model coefficients displayed as red and blue, respectively.

*Figure S5 – Diagram showing data processing workflow for sample-specific perturbation networks*

Workflow for building sample-specific perturbation networks from multimodal datasets. Linear mixed-effects models are used to regress out influence from Age, Sex, and Height. Residuals are used to build background network and sample-specific network distributions which, in turn, are used to build sample-specific perturbation networks. Sample-specific perturbation networks and phenotype categories are used to determine structure of HEC models and these sub-models are optimized using the *Clairvoyance* feature selection algorithm. *Clairvoyance* selects the most discriminative edges and the fitted sub-models are used to build aggregate networks. Aggregate networks are used as scaffolds to build sample-specific perturbation networks.

Figure S6 – Connectivity profiles ***AN_y1_*** for *Otu000281* and molar-excess soluble leptin

Bar chart representing connectivity values within ***AN_y1_*** weighted by logistic regression coefficients.
